# Gram-positive nosocomial infections in a general ICU: emerging new clues

**DOI:** 10.1186/cc9644

**Published:** 2011-03-11

**Authors:** S Milanov, G Georgiev, V Todorova, M Milanov

**Affiliations:** 1 Pirogov Emergency Institute, Sofia, Bulgaria

## Introduction

Gram-positive aerobes are currently the leading cause of infection in many ICUs. Despite this trend, there are still no firm recommendations for empiric Gram-positive antimicrobial coverage in patients with severe nosocomial infections. The current study is an extension of our previous work in this field, aiming to challenge some of the earlier trends and to bring out new clues.

## Methods

A prospective observational study was conducted including all episodes of documented nosocomial infection in a general ICU for a 4-year period (2006 to 2009). Data on demographics, primary diagnosis, co-morbidity, number of indwelling devices, previous microbial isolates and current antibiotics were cross-tabulated according to the presence and type of Gram-positive pathogens. For the identified most likely risk factors, separate contingency tables were constructed and analyzed.

## Results

A total of 339 patients with Gram-positive isolates were identified (51.21% of 662). Gram-positive isolates were more prevalent in patients with obesity (1.27; CI = 1.08 to 1.47) and diabetes (1.28; CI = 1.03 to 1.53). The following independent risk factors for Gram-positive nosocomial infections (RR and 95% CI) were identified: MRSE-gunshot wound (4.18; 2.35 to 5.19), stab wound (4.01; 2.03 to 4.59), polytrauma (1.91; 1.47 to 2.46), previous isolation of both *Acinetobacter *spp. and *Pseudomonas *or *Candida *spp. (2.01; 1.38 to 2.72 and 2.72; 1.71 to 4.21), treatment with aminoglycoside or carbapenem (2.52; 1.59 to 3.42 and 1.37; 1.03 to 1.80); Enterococcus - billiary peritonitis (2.23; 1.27 to 3.73), acute necrotizing pancreatitis (2.23; 1.27 to 3.73), traumatic lesion of urinary bladder with cystostomy (6.68; 3.26 to 9.65), previous isolation of both *Klebsiella *and *Candida *spp. (6.02; 1.85 to 9.40), treatment with cefoperazone + sulbactam or third-generation cephalosporin (3.49; 2.18 to 5.34 and 1.87; 1.17 to 2.92); MRSA - clinical uroinfection (5.27; 1.74 to 13.52), previous isolation of both *Acinetobacter *and *Pseudomonas *spp. (4.21; 1.79 to 9.42); MSSE - treatment with first/second/third-generation cephalosporin ± metronidazole (5.88; 1.84 to 17.16 and 4.65; 1.71 to 12.18); Streptococcus - pelvic inflammatory disease (5.10; 1.35 to 15.75), soft tissue infection (8.32; 2.73 to 45.36), treatment with quinolones (3.45; 1.34 to 8.54).

## Conclusions

New light was shed on the identification of associated risk factors for Gram-positive nosocomial infections in our ICU. Sufficient data were gathered to aid empirical antibiotic choice in such high-risk patients.

